# Women’s experiences of the injury, recovery and desire for rehabilitation after a second-degree vaginal tear—a qualitative study

**DOI:** 10.1007/s00192-021-04951-3

**Published:** 2021-08-09

**Authors:** Clara Daremark, Lina Andréasson, Annelie Gutke, Monika Fagevik Olsén

**Affiliations:** 1 Department of Health and Rehabilitation/Physiotherapy, Institute of Neuroscience and Physiology, Sahlgrenska Academy, University of Gothenburg, Gothenburg, Sweden; 2 Department of Physical Therapy, Sahlgrenska University Hospital, SE 416 50 Gothenburg, Sweden

**Keywords:** Delivery, Injury, Recovery, Vaginal

## Abstract

**Introduction and hyposthesis:**

Eighty-five percent of all vaginal deliveries cause some form of obstetric tear injury. To our knowledge, there are no studies exploring experiences after second-degree tear. Therefore, our study aimed to investigate the experiences of a second-degree vaginal tear regarding aspects of the recovery and need for healthcare and rehabilitation.

**Methods:**

Individual semi-structured interviews were performed and analysed with a qualitative, inductive descriptive approach.

**Results:**

A group of 18 women with a second-degree vaginal tear after delivery were included. Four main categories with associated subcategories were found: (1) feeling uncertainty, with subcategories: not knowing what is normal, concern, confusion and uncertainty regarding pelvic floor muscle training; (2) feeling of security, with subcategories: I have no/I can handle the symptoms, trust in the healthcare system and I have sufficient knowledge; (3) not prioritizing myself, with the subcategories: I cannot find time and others have bigger problems; (4) lack of trust in healthcare providers, with the subcategories: feeling forgotten, not being taken seriously, distrust of the competence of the healthcare providers and resignation.

**Conclusion:**

Women who suffer from a second-degree vaginal tear after pregnancy can feel safe when needs are met but uncertainty is also common when available healthcare and information are perceived as insufficient. The women also feel uncertainty about what is normal after the tear and how to perform pelvic floor exercises.

**Trial registration:**

This trial was registered in “FoU in Sweden” (Research and Development in Sweden). Registration number: 214591.

**Supplementary Information:**

The online version contains supplementary material available at 10.1007/s00192-021-04951-3.

## Introduction

During a vaginal delivery, all structures in the genital area are affected and about 85% of the women develop some form of obstetric injury or perineal tear damage [1–5]. Such tears are graded depending on the extent from 1 to 4 where 4 is the most extensive. A second-degree injury involves the perineum including perineal muscles but not the anal sphincter [6].

To obtain the correct diagnosis of the degree of rupture, digital vaginal palpation and sometimes ultrasound are used [7]. Patients with second-degree injuries are often sutured directly by the midwife but in case of major injuries the suturing is done by an obstetrician [8]. However, there is a risk of tears being underestimated and injury to the anal sphincter missed. Women with a second-degree obstetric injury have been shown to have weaker pelvic floor muscles compared to those with no rupture 6 weeks after delivery [9]. Reduced pelvic floor function may, over time, lead to urogenital prolapse and urinary incontinence [8].

The treatment of incontinence after pregnancy consists of pelvic floor muscle training (PFMT) [1, 10]. Studies have shown that it can be difficult to find the right muscles and 30% of women contract their pelvic floor incorrectly on verbal instruction [11]. An insufficient provision of information from healthcare workers regarding physical changes, pain and pain treatment, PFMT, physical exercises, diet, emotional problems, adaptation to the parent role and childcare has previously been reported by new mothers [12]. A lack of information provided from healthcare workers about the injury and how to treat it has been reported by women with third- and fourth-degree injuries [13]. Importantly, less anal incontinence has been found among patients who have been instructed about PFMT by a specially trained professional [14].

As studies exploring experiences after a second-degree vaginal tear injury are lacking, the aim was to investigate it regarding aspects of the recovery and need for healthcare and rehabilitation.

## Methods

A qualitative method was used to capture patient experiences regarding obstetric tear injuries and the healthcare provided and perceived need for healthcare after delivery.

Primi- and multiparous women who had undergone delivery at Sahlgrenska University Hospital, Sweden, with a second-degree injury were invited to participate in the study after purposive sampling during the inclusion process. Recruitment was done by reviews of patient files and after reports from midwives who had delivered women who had such an injury. Women who did not speak Swedish, or had documented problems with their child, were excluded. Women were recruited until there were three consecutive interviews where no new information emerged.

Current clinical practice in Sweden for women who have a second-degree vaginal injury during delivery is that the injury is sutured in the delivery room, commonly by the midwife. No specific follow-up at the hospitals is offered after discharge but all women are invited to a follow-up by a midwife at their maternity care clinic 6 to 8 weeks after delivery.

The study has been carried out in accordance with the Code of Ethics of the World Medical Association (Declaration of Helsinki) for experiments involving humans. The study protocol was approved by the Regional Ethics Committee for the region of Västra Götaland, Sweden (registration number: 438–16). All participants received oral and written information before the start of the interview and gave their written consent.

Eighteen women agreed to participate in the study. The interviews took place 3 to 4 months after the delivery at the participants’ chosen location, in the hospital or at the home of the participants. The two interviewers (CD and AD) were not involved in any of the care of the women who participated. All researchers are physiotherapists with special interest in women’s health. If questions about the injury and recovery from it were raised during the interview, the women received information after the interview or were referred to the midwife at their maternity clinic.

All interviews started by asking the same question: “Would you like to tell us about your injury in conjunction with the delivery?” The interviews were based on an interview guide; see appendix.

The interviews were recorded using audio-recording programs on two different mobile phones and then stored on a USB memory device. The interviews lasted 15 to 30 min and were transcribed verbatim and analysed.

The content analysis by Graneheim and Lundman was used [15]. The analysis and coding were discussed until a consensus was reached.

## Results

Eighteen participants aged 26–39 years were interviewed in 2017 and 2018. The participants included both primiparous and multiparous women, some of whom had been to a postpartum check-up with their midwife.

The analysis led to 4 main categories with 13 subcategories, presented in Table 1. The categories cover a broad perspective of experiences, some of which are contradictory. Some of the women expressed that the injury and recovery have been troublesome while others had not even understood that they had an injury to such a degree.

**Table 1:** Overview of the division of main categories and subcategories

Main category	Subcategory
Feeling of uncertainty	Not knowing what is normal
	Concern
	Confusion
	Uncertainty regarding pelvic floor muscle training
Feeling of security	I have no/I can handle the symptoms
	Trust in the healthcare system
	I have sufficient knowledge
Not prioritizing myself	I cannot find time
	Others have bigger problems
Lack of trust in the healthcare	Feeling forgotten
	Not being take seriously
	Distrust in the competence of the healthcare providers
	Resignation

### Feeling of uncertainty

The participants felt that they had insufficient knowledge about the injury. Not knowing what they could expect or what they had been through created uncertainty. They found it difficult to know whether they had or had had enough knowledge because they did not know what to expect. This category consists of the following subcategories: not knowing what is normal, concern, confusion and uncertainty regarding the pelvic floor muscle training.

#### Not knowing what is normal

The participants described that it was difficult to know what to expect after a tear injury and which symptoms were normal. They described that it was hard knowing whether symptoms were because of the tear or the delivery. They had accepted the situation because they did not know what was normal or when to seek help.

*“I had so much pain for two weeks, I could barely go out with the dog. I could barely walk to the kitchen. So yes, I didn’t really know what was normal.”*

#### Concern

This subcategory contains experiences regarding concern and fear of or due to their situation. The participants described that they felt anxious about the future and about the injury itself. Some participants described that the lack of information could create fear, others that a lot of information can lead to concern and fear of childbirth.

*“I had read a bit online that this could happen, but I hadn’t…I have good self-preservation skills, so I didn't read too much, because I think if you don't end up being affected, then it’s dumb to read too much and scare yourself unnecessarily…It’s better that if it happens, you deal with it then.”*

#### Confusion

Some participants said that they had been confused when they realized that they had too little knowledge about the injury they had suffered. It was difficult to find out where to turn with their questions, and they felt unsure about whether their questions were adequate. Some participants were confused to find out that they even had had a tear injury as they had not experienced any trouble or pain.

*“When you don’t know, you don’t know what to ask for. It maybe sounds silly. I didn’t know that I was going to have problem with this because I felt so well then.”*

#### Uncertainty regarding pelvic floor muscle training (PFMT)

The participants felt insecure regarding their PFMT. They knew that it was important to do it but found it difficult to know whether they did the exercises correctly. Some participants felt that they had recovered and had no symptoms, but they were still worried about their pelvic floor muscles and developing symptoms.

*“But it’s not always so easy to know if you have found the right muscles or if you’re just tensing your stomach muscles.”*

### Feeling of security

The participants described that they felt well and that they could handle their situation, which created a sense of security. They also described that they had enough knowledge and had received good support from the healthcare system. The subcategories are: I have no/I can handle the symptoms, trust in the healthcare system and I have sufficient knowledge.

#### I have no/I can handle my symptoms

In this subcategory the participants described how they handled the situation regardless of the presence of symptoms. The participants described that they felt confident in everyday life and during physical exertion. In addition, they described a feeling of wanting to be left alone after delivery.

*“Well it felt ok, sort of…I had no specific questions. I knew obviously that it'd be a bit sore. And it was.”*

#### Trust in the healthcare system

The participants were satisfied with the healthcare they received and experienced good support on follow-up midwife visits at the maternity care centre. They also described that they had a feeling of receiving help and the right healthcare when needed.

*“I think I would turn to the midwife first, then they can refer me on to someone else, if needed.”*

#### I have sufficient knowledge

The participants described having sufficient knowledge, and even though they were aware that more information is available, they did not need of it. They knew where to turn to get the right help and where to search for more information. They felt that they had received good information at the obstetric ward but, at that time, they were unable to take it in. They were content with not having received too much information and that they themselves were able to search for the right information as needed.

*“I don’t think I missed any information, but I don’t know if I received any. You find your own information nowadays. You don’t need someone to tell you everything nowadays.”*

### Not prioritizing myself

The women expressed that they were informed and knew what to do but, now being a parent decreased their ability to focus on themselves. It was an active choice not to prioritize themselves. The women compared the problems they experienced with the problems of other women, and when they found their own problems were less, they felt that they did not want to ‘take’ care from others in greater need. The category consists of two subcategories: I cannot find time and others have bigger problems.

#### I cannot find time

The participants described that they found it difficult to prioritize themselves after delivery. The participants also described that they are aware of the importance of PFMT but found it difficult to fit it into everyday life.

*“…before you have children it is a lot easier to focus on yourself but after having a child it’s like, you come last and sure, you can do pelvic floor exercises while you are breastfeeding but, well, it’s just not a priority.”*

#### Others have bigger problems

They also described that they feel that the healthcare system is for those with really big problems and are therefore reluctant to seek help for themselves.

*“It feels like there are so many people who have bigger problems who need help. It's not like when going to someone, like a PT (personal trainer) or someone private where you pay yourself. It's the public healthcare system. And if you don’t have too much trouble then, I’m hesitant to seek care, because their resources should be used for the right things somehow…*”

### Lack of trust in the healthcare system

The participants described feelings of not having received enough support or information from the healthcare system. It was both a sense that support was lacking when they sought help and that they did not receive the right help from the start. This category has the following subcategories: feeling forgotten, not being take seriously, distrust in the competence of the healthcare providers and resignation.

#### Feeling forgotten

The participants described a feeling of being forgotten and under-prioritized by the healthcare providers after delivery. They also described an impression that there were a lot of focus on the child but not on the actual perineal injury or the aftercare of the mother.

*“So, there is a lot of focus on the mother's health when you are pregnant and a lot on the baby's, of course, but when you deliver the main focus is on the baby, and then later it feels like you need to look after yourself now.”*

#### Not being taken seriously

When the participants sought healthcare for their symptoms, they felt that they were not always taken seriously or did not receive adequate answers. They experienced that the healthcare providers diminished their problems, that they were not serious enough/worth seeking care for and they hesitated to seek help.

*“No, I didn't really feel that she was listening or that she was, like, taking it seriously. I don't even know if it should be taken seriously either.”*

#### Distrust in the competence of the healthcare providers

The women experienced that it was difficult to find out where to turn with their questions and what information they could trust. Sometimes the information given was unclear or inadequate. Ignorance about professional competencies among healthcare providers led to a distrust of the care that was offered.

*“I thought why should I see a physiotherapist? It felt kind of wrong, the wrong area of the body. I’ve been to a physiotherapist before and we have focused knees, stuff like that. Now the problems were more private and that felt wrong. If I'd known that this physio is an expert on the muscles in that area, then I would probably have thought differently.”*

#### Resignation

This subcategory contains experiences dealing with participants who finally gave up. The participants felt resignation when they did not know how and what to do or when they did not receive any answers to their questions.

*“So, I called again [...], no it’s nothing strange, you can bleed [laughter]…. …then I gave up a little and felt like, whatever, it seems like you can bleed for any length time, any amount without anyone doing anything. So, I just didn’t care really, I just did put up with it.”*

## Discussion

A second-degree obstetric perineal tear at delivery is believed to have minor impact but the results of this study indicate that the experiences are diverse. Some participants experienced a sense of uncertainty and that neither themselves nor the healthcare providers were sufficient after having suffered a second-degree obstetric perineal tear. On the other hand, others described a sense of security. The experiences of recovery were influenced by a sense of uncertainty when not knowing what is normal and having insufficient information about what to expect. For others there was a sense of security when needs were met, which is in line with previous studies [12]. The women with the injury had different levels of knowledge of the risk of injuries during a delivery and they therefore had varying expectations. Both positive and negative aspects were captured in the material, as the group is heterogeneous and possibly mirrors the situation in society.

How participants viewed the need for healthcare and physiotherapy was divided. Some participants reported that they had not even been aware of having suffered an injury. They found it difficult to express their thoughts when they felt that they did not have enough knowledge about what healthcare is available and what symptoms are normal after a tear injury. The information given in hospital was experienced as insufficient and difficult to understand, in line with a previous study focused on women who suffered a third-to-fourth degree injury [13]. This indicates that information is important regardless of the extent of the injury to increase the feeling of security.

The participants felt as if they had been forgotten and described it as if they had been ‘thrown out’ after delivery and, from then on, they would have to cope on their own. The participants further described the feeling of not being taken seriously when they sought help and how they then eventually gave up, which we interpreted as resignation. The fact that the focus is on the baby and not on oneself during childbirth could be a reason why information had not been taken in or was forgotten. However, information about injuries is important, and it is the responsibility of the healthcare system to provide this information since they have the knowledge about whether an injury exists and its extent.

The main category, “feeling that neither I nor the healthcare system is sufficient,” was partly about experiences of women not receiving enough support from the healthcare system, but also partly about them not knowing what they they should have done. Some women felt somewhat ashamed about not having performed PFMT even though they knew about it—that they did not do everything they could do, potentially due to lack of sleep, low motivation and lack of time. Proper information about the importance of PFMT and individually tailored programs may increase the motivation of new mothers.

Contrary to the feeling that the individual and the healthcare system were insufficient, some participants expressed feelings of security. This was evident by the fact that the participants felt that they trusted the healthcare system and that midwives could give them the right information. It was also evident that the participants, although aware that they did not have a lot of knowledge, still felt that they had sufficient knowledge. Feeling secure can be interpreted as related to whether a woman has received information that matches her specific needs and desires. However, there may be a false sense of security if there are no or minor symptoms. Some consequences of pregnancy and delivery such as urinary or faecal leakage can occur later in life and this may be prevented to some extent by PFMT [8].

The participants’ understanding about the information they were given varied. There were participants who wanted more information before childbirth, others after, some both before and after and participants who did not want more information at all. Common to the participants who had wanted more information was that they wanted more information about the injury itself, what was normal and what to expect when leaving hospital. The possible dilemma between information that provides security or is scary could be met with person-centred care, a philosophy-based management where the person’s needs and resources direct the intervention [16].

Some participants described how they, during the follow-up visit at the maternity care centre, found out that they were not performing the PFMT correctly and did not know how to do it. Vaginal digital palpation has been shown to be important to provide feedback and individualized pelvic floor exercises [11]. It is therefore important that physiotherapists incorporate this in the follow-up protocol.

The participants had adapted their needs to what was offered within the healthcare system without reflecting on whether it was enough in both a short- and long-term perspective. It is known that some problems related to giving birth do not start until later, most often at menopause [10]. Therefore, there should be more healthcare for women after delivery to prevent symptoms from developing later in life. The result of this study may be useful for all healthcare professionals who meet mothers after a recent delivery to gain an increased understanding of experiences after a tear injury. The study suggests that physiotherapists could take a larger part in post-natal care by providing person-centred information about the pelvic floor and tailored exercises.

One limitation of the study is that the participants were limited to one hospital clinic. To improve the transferability, participants from different hospitals and healthcare regions could have been included. However, the obstetrical care was only provided by midwives and the transferability to care by obstetricians is not known. To further improve the credibility of the study, two of the authors had no specific experience with postpartum patients. To improve dependability, i.e., the risk of changes in analysis decisions over time due to for example researchers’ learning, an open dialogue within the research team was undertaken to increase the trustworthiness of the study. Another limitation is that the participants were all Swedish speakers. Linguistic limitations may affect the experience after a perineal injury such as how information is perceived and how that influences the feeling of security. The results of the study have been presented as a whole and not according to how many who expressed what, according to the guidelines [15]. Regardless, a quantification could have added what the women emphasized.

In conclusion, women who suffer from a second-degree vaginal tear after pregnancy can feel safe when needs are met but uncertainty is also common when available healthcare and information are perceived as insufficient. The women feel uncertainty about what is normal after the tear and how to perform the pelvic floor exercises. A physiotherapist could be the profession that provides individualized information and rehabilitation related to the pelvic floor.

### Supplementary Information

ESM 1 (DOCX 21 kb)

## Appendix 1: Interview guide

Experiences of the vaginal injury and suffering from it.

- Description of the injury.
- Experiences of having the injury.
- Experiences of symptoms of and after the injury.

Experiences of the care of the injury at the hospital

- Healthcare and nursing in general
- Information
- Examinations
- Interventions

Experiences with the healthcare system after discharge

- Contact with the healthcare system regarding the symptoms after discharge
- Experiences of seeking healthcare for the symptoms after discharge

Opinions about information regarding the injury

- Description of sources of information about the injury
- Desire for knowledge about the injury before and after delivery
